# Robotic-Assisted Thoracoscopic Surgery Versus Video-Assisted Thoracoscopic Surgery: Which Is the Preferred Approach for Early-Stage NSCLC?

**DOI:** 10.3390/jcm14093032

**Published:** 2025-04-28

**Authors:** Nikolaos Syrigos, Eleni Fyta, Georgios Goumas, Ioannis P. Trontzas, Ioannis Vathiotis, Emmanouil Panagiotou, Nikolaos I. Nikiteas, Elias Kotteas, Dimitrios Dimitroulis

**Affiliations:** 1 Hellenic Minimally Invasive and Robotic Surgery (MIRS) Study Group, Medical School, National and Kapodistrian University of Athens, 11527 Athens, Greece; 2 3rd Department of Internal Medicine, Oncology Unit, “SOTIRIA” General Hospital of Chest Diseases, National and Kapodistrian University of Athens, 11527 Athens, Greece

**Keywords:** video-assisted thoracoscopic surgery [VATS], robotic-assisted thoracoscopic surgery [RATS], non-small cell lung cancer [NSCLC], minimally invasive surgery

## Abstract

Lung cancer is the leading cause of cancer-related mortality among both men and women worldwide, underscoring the need for an effective treatment strategy. For early-stage non-small cell lung cancer [NSCLC], surgical resection is the standard treatment. Robotic-assisted thoracic surgery [RATS] and video-assisted thoracic surgery [VATS] are better than open thoracotomy because they are less invasive. Recent lung cancer screening programs are detecting NSCLC at earlier stages, which is expected to result in an increase in the number of NSCLC surgeries as early-stage cases are diagnosed. A limited number of randomized controlled trials have compared RATS and VATS in operable NSCLC. We conducted a literature review to summarize the available evidence on these two surgical techniques. The purpose of this study is to compare the intraoperative and postoperative outcomes of RATS and VATS in early-stage NSCLC patients. RATS shows lower conversion rates to thoracotomy (6.3% vs. 13.1% *p* < 0.01) and more thorough lymph node dissection than VATS (e.g., 7.5 vs. 5.6 stations, *p* < 0.001). However, RATS is linked to considerably higher costs (USD 22,582 vs. USD 17,874, *p* < 0.05) and longer operative times (median 241.7 vs. 214.4 min, *p* = 0.06). The two techniques exhibited minimal differences in postoperative complications and pain, while RATS patients experienced shortened hospital stays (4–5 vs. 5–6 days, *p* < 0.006). While the accuracy of staging and treatment planning is improved by the improved lymph node retrieval in RATS, the long-term survival rate is still uncertain.

## 1. Introduction

Lung cancer is the leading cause of cancer-related mortality and the second most prevalent cancer worldwide [1]. Based on histological classification, lung cancer is classified as non-small cell lung cancer [NSCLC] and small cell lung cancer [SCLC]. A clear understanding of the relative advantages and disadvantages of robotic-assisted thoracoscopic surgery [RATS] and video-assisted thoracoscopic surgery [VATS] is paramount among healthcare providers, patients, and healthcare systems.

This would allow for an informed decision on treatment and, therefore, the best outcomes for the patients. With their increasing use, both RATS and VATS are still being debated regarding which technique is superior in safety, effectiveness, and cost-effectiveness. We compared the effectiveness of minimally invasive surgical techniques in patients with early-stage NSCLC undergoing either VATS or a robotic approach.

NSCLC accounts for 85% of all lung malignancies and represents the most frequent cause of cancer mortality in individuals >50 years of age [2]. Two main factors have led to a bigger rise in survival rates in NSCLC: low-dose spiral CT scanning for lung cancer, which helped find the disease earlier [1,2,3], and the arrival of newer treatments like targeted therapies and surgery [2]. Surgical resection is still considered the standard of care for early-stage NSCLC and should be performed as soon as possible [4]. For these patients, common surgical procedures include lobectomy, segmentectomy, or pneumonectomy. Recently, RATS and VATS have emerged as popular, minimally invasive alternatives to traditional open thoracotomy [5,6,7].

### 1.1. Definition and Description of VATS

In the past 20 years, VATS lung cancer resections have become very popular and are still a well-known minimally invasive alternative to open lobectomy for early-stage NSCLC [8,9]. The total number of cancers resected by VATS has increased significantly recently [10]. VATS utilizes a small video camera, which is introduced into the patient’s chest via a scope. The procedure provides the surgeon with a much better view of the anatomy and pathology as compared to conventional thoracotomy. In total, 1–4 ports are made in the chest for the insertion of instruments with or without rib spreading, allowing the surgery to be performed under direct vision. This necessitates considerable expertise and patience, as VATS conventionally depended on a two-dimensional perspective of the three-dimensional anatomy, resulting in a more challenging learning curve. However, the utilization of a 3D camera system in VATS, which enhances surgical precision and depth perception, reduces the gap with RATS. Compared to open surgery, VATS lobectomy tends to be less morbid [8]. Multiple studies have indicated that VATS is associated with a reduced rate of postoperative complications, reduced pain, less hemorrhage, shorter hospital length of stay, and quicker recovery compared to open thoracotomy [6,7,8,11,12]. Elderly patients with several comorbidities who are at increased risk for thoracotomy commonly undergo VATS [13]. These same individuals, at higher risk for thoracotomy, also have an increased likelihood of experiencing perioperative complications, especially those related to pain and compromised pulmonary function. Consequently, VATS may provide an added advantage to this category of patients. Although VATS is a well-established minimally invasive surgical technique, it is critical to note its limitations that may negatively impact lobectomy procedures. These are the limited flexibility of the instrument and a disrupted eye–hand–target axis. The limitations and technical demands of VATS led to the development of RATS.

### 1.2. Definition and Description of RATS

RATS was first introduced into clinical practice in 2002 and has since become a significant tool in the surgical management of NSCLC. Several studies have proved its safety and feasibility, hence confirming its value in the surgical management of NSCLC [5,12]. The robotic arms controlled by the surgeon in RATS are more precise and dexterous, allowing refined movement during the surgery. In addition, the system is designed with 3D visualization, offering the surgeon a much clearer and more detailed view of the operating area, hence improving surgical outcomes and reducing complications. RATS enables surgeons to do accurate, minimally invasive procedures using small incisions in the body. Consequently, RATS is seen as a method replacing traditional open surgery in lung cancer patients. There is evidence to show that RATS, as a newer approach compared to VATS, has many advantages for NSCLC patients. The most significant reported advantage is that RATS has a high level of precision and dexterity. Since the robot system allows the surgical tools to mimic the surgeon’s hand movements within the patient’s body, there is a substantial improvement in their range of motion compared to conventional manual thoracoscopic instruments. This makes it easier to work with tissues and blood vessels while minimizing the risk of damage. This is especially important during lung cancer surgeries because many important blood vessels are close by. In spite of the possible benefits that robotic technology could bring to thoracic surgery, it is not widely used because there is a lack of high-quality prospective data, and RATS procedures are more expensive than VATS procedures.

### 1.3. Differences in Surgical Equipment

Modern RATS systems, such as the Da Vinci robotic system, address several drawbacks of VATS. A robotic system has cutting-edge three-dimensional optics that allow for high-definition visualization in three dimensions, a real-time view of the surgical field, and no tremor, which makes up for the lack of tactile feedback that is common in minimally invasive techniques [5,12,14,15,16]. Consequently, the learning curve for robotic surgery appears to be less steep and quicker than that of laparoscopic surgery, reducing surgical fatigue [16,17]. Its flexibility and stability enable surgeons to perform minimally invasive surgery with greater ease, especially in complex intrathoracic procedures [16]. Thus, RATS systems may help to improve the visualization of sub-lobar lesions and aid in the identification and preservation of small sub-lobar vessels [15]. In contrast, VATS is constrained by non-ergonomic rigid instruments and the absence of an eye–hand–target axis, resulting in a particularly challenging dissection within a confined space. The long learning curve and technical demands associated with VATS may prevent its widespread acceptance and are typically performed mainly by proficient and highly skilled surgeons [3].

## 2. Materials and Methods

A systematic search of the literature from 2005 to 2024 in PubMed and Embase was used to compare RATS and VATS for early-stage NSCLC. To minimize selection bias, studies were included if they directly compared RATS and VATS for early-stage NSCLC and reported on at least one of the following outcomes: surgical (operative time, conversion rates, complications), oncologic (lymph node dissection, R0 resection rates), or economic (hospital costs, length of stay). Meta-analyses, randomized controlled trials [RCTs], and large observational studies were selected for high-quality evidence. We did not include case reports or editorials, studies that focused on locally advanced or metastatic NSCLC, or studies that did not give separate data for RATS and VATS.

Although no formal heterogeneity analysis was conducted, we evaluated key sources of variability, including surgical methods and research design. We collected data on the practicality of the surgical tools used and parameters observed pre- and post-operatively to compare the two procedures. Intraoperative factors include surgical duration, conversion rate to thoracotomy, lymph node extraction, and intraoperative bleeding. Postoperative variables include complications, drainage duration, postoperative bleeding, pain intensity, quality of life, hospital stay duration, mortality rates, readmission frequencies, and financial aspects. Differences in surgical equipment, administration of neoadjuvant chemotherapy, and long-term survival were also analyzed.

While conducting the results analysis, we assessed these discrepancies and their potential impact on the reported outcomes. *p*-values, confidence intervals, and survival estimates (hazard ratios) were included when applicable to evaluate the statistical significance and long-term oncological outcomes. Search terms used were “robotic-assisted thoracic surgery”, “video-assisted thoracoscopic surgery”, “NSCLC”, and “minimally invasive surgery”. Institutional ethics approval was not needed; the data used in this retroactive review were publicly available.

## 3. Results

### 3.1. Intraoperative Complications

#### Operation Time

More time consumed by RATS remains a concern for widespread adaptation of the system to any early-stage NSCLC cancer. According to the study conducted by Park et al., the median operative time for RATS was 3.5 h [18] (Table 1). Swanson et al. conducted a retrospective study, revealing that operating times for RATS lobectomy exceeded those of VATS lobectomy (4.49 h vs. 4.23 h) and wedge resection (3.26 vs. 2.86 h), with statistical significance (*p* < 0.01) [19]. Augustin et al. found that RATS procedures took longer than VATS procedures (215 min vs. 183 min, *p* = 0.036) [20]. Deen et al. reviewed 184 consecutive operations and found that the robotic group had a longer operating room time (223 min) compared to the VATS group (202 min), with statistical significance (*p* = 0.04) [21]. Oh et al. also revealed significant findings in their series; the mean operating room time for RATS was higher by 28 min compared with VATS (*p* < 0.01) [22]. More studies that looked back at the data confirmed that operations took longer in the RATS group (*p* < 0.001) [23,24]. Qiu et al., on the other hand, found that the robotic sleeve technique for centrally located NSCLC had the shortest operating time compared to other methods, including VATS (*p* < 0.01) [25].The BRAVO trial, a randomized clinical trial, found that RATS took a little longer to perform than VATS, but the difference was not statistically significant (241.7 min vs. 214.4 min) [26]. Mao et al.’s meta-analysis found no statistically significant difference in operative duration between RATS and VATS [27].

### 3.2. Conversion Rate to Thoracotomy

Augustin et al. presented a retrospective study that found no statistical difference in the conversion rates between RATS and VATS lobectomies [20], which was confirmed afterward by the meta-analytical review performed by Mao et al. (odds ratio [OR] = 1.42, 95% confidence interval [CI]: 0.70–2.88, *p* = 0.33) [27]. On the other hand, Oh et al. found that RATS had a much lower rate of surgical conversion than VATS (13.1% vs. 6.3%; *p* = 0.0001). In comparison, the VATS group had a conversion rate of 5.9% [22], whereas Qiu et al. found a 0% conversion rate for open surgery in the robotic group [25].

### 3.3. Lymph Node Dissection

Lymph node dissection [LND] is an essential part of the surgical treatment of early-stage NSCLC. It is essential for accurate staging and patient prognosis and vital when considering adjuvant treatment. Regarding LND, both the number of lymph node groups and the total quantity of lymph nodes dissected should be considered. We note that, in the VATS era, LND may not be as extensive or accurate as open surgeries. This is partly due to the limitations of the VATS technique and the surgeon’s skill in retrieving all nodes, which, in certain cases, may necessitate conversion to open surgery for completion. RATS is perceived to enable surgeons to carry out more accurate and extensive LNDs when compared to VATS methods, as well as open surgery.

Veronesi et al. conducted a comparison between RATS and VATS in terms of lymph node evaluation and found that RATS was associated with a higher number of removed lymph node stations (6, interquartile range [IQR] 4–6 vs. 4, IQR 3–5; *p* = 0.0002) as well as an increased number of mediastinal (7, IQR 5–10 vs. 5, IQR 3–7; *p* = 0.0001) and hilar lymph nodes (7, IQR 5–10 vs. 4, IQR 2–7; *p* = 0.0003) [28]. Nasir and colleagues stated that surgeons can achieve R0 resection (complete tumor resection with no microscopic residual disease [R0]) in lung cancer by ensuring complete and accurate lymph node dissection [29]. In line with these findings, Zhang et al. reported that RATS yielded a significantly higher number of lymph nodes with a median of 11 (IQR 10–13) total lymph nodes compared to 6 (IQR 5–7) in the VATS group (*p* < 0.001) [30]. Similarly, a study by Novellis et al. revealed that the number of lymph node stations removed was significantly greater in the RATS group (mean, 4.7) in contrast to the VATS group (mean, 2.7) (*p* < 0.001) [12]. Runsen Jin et al. said that RATS had better results than VATS in lung cancer surgery. This was shown by a higher number of lymph nodes being removed [IQR, 8–15 vs. IQR, 8–13; *p* = 0.02] and a more accurate assessment of the pathological N-stage [31]. The results of the meta-analysis by Mao and colleagues showed that there was no significant difference between the two groups in the number of lymph node dissections (standardized mean difference [SMD] = 0.31, 95% CI: −0.09–0.71, *p* = 0.13) [27]. However, RATS demonstrated superiority in the meta-analysis focusing on the total number of lymph nodes dissected (SMD = 0.31, 95% CI: 0.13–0.49, *p* = 0.001) [27]. In a study by Qiu et al., it was shown that, for centrally located NSCLC, the average number of lymph nodes harvested during robotic sleeve and VATS procedures was similar. There were no statistically significant differences in the pathologic T stage or pathologic N stage [25].

However, the potential survival benefit obtained from acquiring another LN and nodal station in the RATS group, though essential for exact staging and treatment preparation, remains doubted in terms of its impact on long-term survival. A recent study by Dezube et al. indicated that the optimal number of lymph nodes excised for this surgery was four, with more resections yielding no enhancement in survival rates [32]. Hennon et al. performed a study comparing various surgical techniques: RATS, VATS, and open thoracotomy. The average number of lymph nodes harvested was much higher with the open thoracotomy method compared to other methods, and the rates of upstaging were higher (*p* < 0.01) [33]. In fact, they found that both RATS and VATS were linked to higher survival rates compared to open surgery in the multivariable analysis of their study (*p* < 0.01). The results again suggest that nodal upstaging is less likely to be related to improved long-term outcomes. Nevertheless, it is important to note that VATS was linked to a longer overall survival rate for people with stage I disease compared to the open method (hazard ratio [HR] = 0.081, *p* < 0.01), but RATS was not linked to the same outcome [33]. Future studies need to determine whether the enhanced LN dissection seen in the RATS group significantly affects long-term survival.

### 3.4. Intraoperative Hemorrhage

Intraoperative hemorrhage is considered an important factor in determining the preferred approach for early-stage NSCLC surgery because excessive bleeding prolongs surgery time and increases complication risks, thus affecting the results of patient outcomes. The research conducted by Qiu and colleagues revealed that the robotic sleeve approach for centrally located NSCLCs had the least estimated blood loss, with statistical significance (*p* < 0.05) [25]. Augustin et al. highlighted a notable distinction, demonstrating that VATS results in reduced intraoperative blood loss compared to the RATS method, as evidenced by lower postoperative hemoglobin levels [20]. Adams and colleagues reported that, although the mean blood loss during RATS was minimal, no variation was observed in the rate of intraoperative blood transfusions between robotic and VATS techniques [23]. Oh et al. observed no statistically significant difference in the incidence of intraoperative hemorrhage between the two groups (*p* = 0.93) [22].

### 3.5. Postoperative Complications and Hospital Mortality

Numerous studies evaluated postoperative complications and in-hospital mortality. Mao et al. reported that the incidence of postoperative complications was similar in both groups (OR = 0.947, 95% CI: 0.79–1.14, *p* = 0.56). Nonetheless, upon conducting a subgroup analysis based on the publication date of the studies, it was discovered that the literature published after 2015 demonstrated a lower incidence of postoperative complications in the RATS group compared to the VATS group (OR = 0.85, 95% CI: 0.75–0.96, *p* = 0.01 [27]). The ROMAN study did not find any significant differences between the two approaches in terms of perioperative complication rate, conversion rate, and early postoperative outcomes. The ROMAN study found that complications after surgery happened in 13 cases (34% of the robotic group) and 9 cases (23% of the VATS group). However, despite the noted difference, it was not statistically significant [28]. Louie et al. also reported that all postoperative outcomes were comparable between the two groups, including complications and 30-day mortality (RATS 0.6% vs. VATS 0.8%, *p* = 0.4) [24]. Concurrently, Adams et al. showed that postoperative complications and results were comparable between RATS and VATS patients [23]. Deen et al.’s retrospective study, which analyzed 184 consecutive operations, observed no significant differences in complication rates [21]. In the RVlob trial, both groups revealed similar postoperative complications (*p* = 0.45), without any mortality during the perioperative period [31]. Qui and colleagues reported that the 90-day mortality rate in the VATS group was 6.3%, whereas no death occurred in the robotic sleeve lobectomy (*p* = 0.051), with no statistically significant difference in the incidence of each postoperative complication between the two groups [25]. In this study, the median follow-up was 21 months in the robotic and 27 months in the VATS group. The robotic and VATS approaches did not significantly differ in terms of OS or DFS: 3-year DFS rates were 76.3% (95% CI, 0.60–0.88) vs. 65.8% (95% CI, 0.49–0.80), *p* > 0.05 [25].

Using a large database, Oh et al. found that RATS was associated with a reduced overall postoperative complication rate (34.1% vs. 37.3%; *p* = 0.006) and lower 30-day complication rates (37.3% versus 40.5%, *p* = 0.013). The study indicated that RATS had a considerably lower overall complication rate than VATS, particularly for postoperative bleeding and myocardial infarction (4.4% vs. 9.3% *p* < 0.0001 and 0.3% vs. 0.8%; *p* = 0.03, respectively) [22]. Patients who underwent RATS had comparable intraoperative complication rates with the VATS group (3.2% vs. 3.1%, *p* = 0.94) [22]. The study conducted by Kent and colleagues showed that robotic resection was associated with lower mortality (0.2% vs. 1.1%) and similar overall complication rates (43.8% vs. 45.3%) compared to VATS, but none of these findings were statistically significant [6]. In the BRAVO trial, there was a tendency for a lower incidence of postoperative complications within 90 days in the RATS group compared to the VATS group, although this difference did not reach statistical significance. However, this tendency disappeared when focusing solely on major complications [26].

On the other hand, Paul et al. conducted a comparison of perioperative outcomes between patients who underwent robot-assisted lobectomy and those who underwent thoracoscopic lobectomy from 2008 to 2011 (2498 robotic lobectomies; 37,595 VATS lobectomies). Any complication occurred more frequently in patients undergoing robotic-assisted lobectomy than in those undergoing thoracoscopic lobectomy (50.1% vs. 45.2%, *p* < 0.05). Significantly higher rates for robotic patients, particularly cardiovascular complications at 23.3% versus 20.0% and iatrogenic bleeding complications at 5.0% versus 2.0%, were observed (*p* < 0.05). Moreover, the risk of iatrogenic bleeding complications remained higher even after performing multivariable analyses. However, it should be indicated that the above study had numerous limitations and a significant percentage of robotic surgeries were conducted in smaller to medium-sized hospitals, and equal numbers were non-teaching institutions with average patient volumes [34]. Another study by Swanson et al. also disclosed similar results of the two surgical methodologies but disclosed a higher proportion of minor adverse events among those patients who had been subjected to robotic lobectomy. In fact, the odds of a minor event are 4.24 times higher (odds ratio) for lobectomy patients treated with a robot compared to their non-robotic counterparts [19].

### 3.6. Post-Operative Drainage Days

An important consideration when comparing postoperative results between RATS and VATS in early-stage NSCLC is the evaluation of postoperative drainage days. Qui et al. presented a significant finding that highlights the benefits of robotic sleeve lobectomy for centrally positioned NSCLC. They mentioned that the tube drainage time was the lowest in the robotic group when compared with VATS (4.2 days versus 7.2 days, respectively, *p* < 0.01) [25]. Mao et al., on the other hand, found that there was no statistical difference between the VATS and RATS groups in the number of days of postoperative drainage (SMD = −0.02, 95% CI: −0.19–0.15) (*p* = 0.81) [27]. This trend remained stable even after rigorous subgroup analysis. In the Bravo trial, there was no statistically significant difference between the RATS and VATS groups in the median drainage time (2 [1,2] days vs. 2 [1,2,3,4] days; *p* = 0.27) [26]. However, in the Rvlob trial, the patients undergoing RATS exhibited a significantly greater total pleural drainage volume (830 mL [IQR, 550–1130 mL] vs. 685 mL [IQR, 367.5–1160 mL], *p* = 0.007) compared to VATS [31]. These varying trends in observations underline the fine, complex variability in the duration of postoperative drainage between different surgical modalities. Further research and analysis are needed to explain such disparities in causes and to enable a broad understanding of the importance of postoperative drainage duration in optimizing patient outcomes.

### 3.7. Post-Operative Hemorrhage

Both RATS and VATS for early-stage NSCLC can be complicated by postoperative hemorrhage, which must be followed up and treated with due care. Adams et al. reported that the rates of postoperative blood transfusion were significantly lower within the RATS group in comparison to VATS (0.9% versus 7.8%; *p* < 0.002) [23]. Oh et al. found that the VATS group had higher rates of postoperative bleeding while the RATS group had lower rates of needing blood transfusions both during and after surgery [22]. The significance of hemorrhage in postoperative morbidity was further underscored by Louie et al., who stated that it was the most prevalent cause of reoperation in both groups [24]. More VATS patients returned due to hemorrhage than their robotic counterparts [24], a trend which is consistent with other studies [6,9,35] but is not statistically significant. The finding, therefore, raises speculation on the factors involved, perhaps implicating the increased skill of automated tools and the effectiveness of bipolar cauterization with RATS in ensuring better hemostasis when compared to VATS techniques. In fact, these multivariate insights are noteworthy and provide a deeper understanding of the more complex relationship between the type of surgical approach and outcomes in terms of hemorrhage.

### 3.8. Post-Operative Pain and Quality of Life

Another important factor to consider when comparing RATS and VATS is the assessment of postoperative care. The study by Brian E. Louie et al. points to a beneficial advantage of the robotic approach because patients in the robotic group had a shorter duration of post-discharge opioid use, *p* = 0.039, thus enabling them to return more quickly to daily activities (*p* = 0.003) [36]. The study conducted by Oh and colleagues revealed that patients who underwent RATS were more likely to be discharged home (92.7% vs. 90.9%, *p* = 0.0108) rather than to transitional healthcare facilities [22]. However, the study by Paul et al. revealed that robotic-assisted lobectomy reflects lower rates of routine discharge as compared to conventional lobectomy (60.8% vs. 70.3%, *p* < 0.001). These findings led to an increased likelihood of being discharged into healthcare facilities rather than returning home (*p* = 0.6) [26,34]. The BRAVO trial conclusively demonstrated that there was no statistically significant difference between RATS and VATS in terms of postoperative pain during the first 3 days and 30 days following lobectomy. The evaluation of postoperative analgesic needs at the 30-day mark also indicated no statistically noteworthy differences [26].

### 3.9. Hospital Length of Stay

According to Louie et al., the median length of hospital stay was four days for both groups. However, a higher percentage of patients who underwent robotic lobectomy were discharged earlier, with an interquartile range of 2–5 days, compared to 3–6 days for VATS lobectomy (*p* < 0.001) [24]. The in-hospital mortality rates for patients who underwent RATS and VATS lobectomy were 0.3% and 0.6%, respectively, with no statistically significant difference (*p* = 0.18). The 30-day mortality rates for robotic lobectomy and VATS lobectomy were 0.6% and 0.8%, respectively, and once again there was no statistically significant difference between the two groups (*p* = 0.42) [24]. According to a study by Oh et al., the median hospital stay for RATS was significantly shorter than that for VATS (5 days vs. 6 days, respectively, *p* < 0.006). However, the postoperative mortality rates were similar for RATS and VATS (*p* = 0.44) [22]. Mao et al. found that there was no notable difference in the length of postoperative hospital stay between the RATS group and the VATS group (SMD = 0.003, 95% CI: −0.10–0.11), *p* = 0.957) and the postoperative hospital mortality between them (OR = 0.72, 95% CI: 0.47–1.11, *p* = 0.139) [27]. In the RVlob Trial, an open-labeled prospective RCT, both groups showed similar perioperative outcomes in terms of hospital length of stay (*p* = 0.76) [31]. Swanson et al. found that the length of hospital stay was comparable for both robotic and VATS lobectomies since the mean hospital stay for both cohorts did not exhibit any statistically significant difference for lobectomy (6.07 vs. 5.83 days; *p* = 0.61) and wedge resection (5.23 vs. 5.38 days; *p* = 0.71) [19]. However, Deen and colleagues reported that, although robotic cases had the shortest length of stay, this finding did not reach statistical significance (*p* = 0.777) [21]. Novellis et al. reported that the median length of stay in the hospital was significantly shorter in the robotic group (4 days) compared with the stay of 5 days in the VATS group (*p* < 0.001) [12].

### 3.10. Hospital Readmission Rate

The BRAVO trial found that the RATS approach resulted in a statistically significant reduction in hospital readmissions for lobectomy-related complications 90 days following surgery. Compared to VATS, the readmission rate following RATS was much lower: 2.7% for one patient against 20.5% for eight patients, with a statistical significance of *p* = 0.029 [26]. The ROMAN study found a trend toward lower readmission rates after RATS, although this trend did not achieve statistical significance (*p* = 0.08) [28]. Additional research is required to better understand the factors impacting postoperative outcomes.

### 3.11. Cost

Robotic-assisted surgical procedures have significantly higher total costs, which pose a significant challenge for both hospitals and insurers. Occasionally, the expenses associated with RATS can become so high that patients who truly require such advanced technology cannot afford the treatment. Veronesi et al. estimated an additional cost of approximately EUR 2000 for each robotic procedure when compared to open surgery, or VATS [37]. We looked at differences in costs using nationwide inpatient sample charge data and found that RATS-lobectomy cost USD 4708 more than VATS-lobectomy (USD 22,582 vs. USD 17,874, *p* < 0.05) [34]. A survey by Swanson et al. demonstrated that for lobectomy procedures, the resection’s average inpatient care cost with RATS was USD 25,040.70 compared to USD 20,476.60 for VATS (*p* = 0.0001). In the case of wedges, the average costs were USD 19,592.40 and USD 16,600.10 for RATS and VATS, respectively (*p* = 0.0001) [19]. In Augustin’s study, the costs of RATS were significantly higher, adding 44% to the costs of a regular VATS lung lobectomy [20]. The RVlob trial showed that the RATS group had much higher hospitalization costs (USD 12,821 [IQR: USD 12,145–USD 13,924] vs. USD 8009 [IQR: USD 7014–USD 9003], *p* < 0.001) [31]. Huang et al.’s study indicated that RATS incurred significantly higher costs than VATS, with figures of USD 85,324.41 ± USD 12,893.44 versus USD 68,733.13 ± USD 14,781.32, respectively (*p* < 0.01) [38].

The analysis by Novellis et al. indicated that the cost of robotic surgery was approximately 13.5% greater than that of VATS. It is noteworthy that the hospital considered the robotic approach to be cost-effective, as it incurred approximately 18% lower costs in comparison to health service reimbursement. The reduced duration of hospital stays for robotic patients demonstrated benefits in resource optimization and decreased waiting times [12]. According to Deen’s study, robotic cases cost USD 3182 more than VATS (*p* < 0.001). This was because of the cost of robotic-specific supplies and capital loss. After removing these factors, no statistically significant difference in procedure cost between the modalities was found [21]. However, a cost-utility analysis determined that RATS is “not cost-effective” for early-stage lung cancer, primarily because of elevated equipment costs [4]. Hopefully, with technological advancements, robotic surgery is expected to become increasingly affordable and accessible, thereby enhancing the benefits of RATS for lung cancer procedures.

### 3.12. Administration of Neo-Adjuvant Therapy

Not many studies have been performed to compare the feasibility and oncological effectiveness of RATS versus VATS after neoadjuvant immunochemotherapy and different points of view are still being discussed. There are a few studies that demonstrate that lobectomy following neoadjuvant treatment does not raise the risk of disease and mortality [39,40,41]. However, choosing neoadjuvant chemoimmunotherapy could make surgery more difficult and increase the risks that come with it, such as the anastomosis not healing properly. Robotic-assisted thoracoscopic surgery is a novel type of surgery that uses the da Vinci Surgical System to perform operations, allowing surgeons to perform precise, minimally invasive surgery on small incisions on the body. Consequently, this type of surgery is particularly useful for early-stage patients with NSCLC, as the limited stress and trauma to the patient could mean that postoperative chemotherapy and radiation therapy may also be feasible [42].

In their study, Romero et al. discovered that about 20% of NSCLC patients who had VATS as their first surgery needed to be switched to open thoracotomy. This rate was much higher in patients who received neoadjuvant chemoimmunotherapy than in those who did not [43]. According to the TOP1201 clinical trial, 25% of patients who had VATS after neoadjuvant chemoimmunotherapy needed to switch to thoracotomy [44]. On the other hand, the rate of conversion to thoracotomy for RATS after neoadjuvant chemoimmunotherapy was only 4.5%. No correlation was observed with perioperative morbidity or mortality, signifying a notable degree of safety [45]. The retrospective study conducted by Hanbo Pan et al. compared the efficacy of RATS versus VATS in managing NSCLC patients who underwent neoadjuvant immunochemotherapy. Their analysis revealed no discernible disparities in surgical outcomes, pathological findings, or postoperative complications between the two techniques (all *p* > 0.050). Additionally, they noted a comparable one-year relapse-free survival (RFS) rate of 82.96% for RATS and 85.23% for VATS, with a *p*-value of 0.8 [46].

Zeng et al.’s study, where data were collected from 220 people who had been diagnosed with NSCLC and had thoracic surgery after neoadjuvant chemoimmunotherapy. The patients in this cohort were stratified into two groups: those who received VATS and those who received RATS procedures. The study findings indicated that patients who underwent RATS achieved superior results on most key parameters compared to those who underwent VATS [47]. A lower conversion rate to thoracotomy was observed in RATS patients, alongside improved pain scores and more lymph node stations harvested: VATS versus RATS, 28.2% versus 7.5%, *p* < 0.001; *p* = 0.002; 5.63 ± 1.75 versus 8.09 ± 5.73, *p* < 0.001. Lymph node removal occurred more frequently in RATS patients; however, the overall complication rates were comparable between the two groups.

### 3.13. Long-Term Survival

The existing literature lacks substantial data regarding the comparison of survival outcomes between robotic and VATS lobectomy in NSCLC patients. The retrospective analysis conducted by Wilson et al. revealed significant data about oncologic efficacy. They documented a 2-year overall survival rate [OS] of 87.6% for RATS [48], which is favorably contrasted with the previously reported 5-year OS rate of 80% by Park and colleagues [18]. Remarkably, RATS exhibited a disease-free survival rate [DFS] of 70.2% for operable NSCLC [18]. In contrast, VATS demonstrated less favorable outcomes, with a 5-year OS rate of 76.5% for early-stage NSCLC and a DFS rate of 60% [49]. The study conducted by Wilson et al. lacked a RATS control group; nonetheless, a meticulous comparison was performed against a recently published series.

Fabri et al. studied 619 patients with primary lung cancer who underwent robotic thoracic surgery or VATS. Patients who received robotic lobectomy had significantly greater DFS and lower recurrence rates than those who underwent VATS: the 3-year DFS was 92.4%, and the 5-year DFS was 90.3%, compared to 77.6% (*p* < 0.001). No substantial difference was observed in OS outcomes between the two surgical approaches (3-year OS: 75.9% vs. 82.3%; 5-year OS: 70.5% vs. 68.5%; *p* = 0.63) [50]. Similarly, the retrospective study conducted by Qui et al. revealed that there were no significant differences in OS and DFS between the robotic and VATS groups [25], with 3-year DFS among the robotic and VATS groups 76.3% (95% CI, 0.60–0.88) vs. 65.8% (95% CI, 0.49–0.80). Hanbo Pan and colleagues conducted a retrospective study to evaluate the long-term efficacy of minimally invasive surgery in patients with NSCLC aged 35 years or younger. In the RATS group, the 5-year RFS rate was 87.0%, while the OS rate was 95.9%. The VATS cohort demonstrated an OS rate of 95.7% and a 5-year RFS rate of 86.2% (all *p* > 0.05). Notably, the RFS and OS profiles of both surgical approaches were comparable [46].

**Table 1:** Detailed comparative table by study: RATS vs. VATS for early-stage NSCLC.

Outcome	RATS	VATS	*p*-Value	Interpretation	Reference
Lymph Node Dissection	7.5 ± 1.75 stations	5.63 ± 1.75 stations	<0.001	RATS provides more extensive lymph node dissection.	Zeng et al. (2023) [47]
	5.2 ± 1.4	3.9 ± 1.2	0.0001		Veronesi et al. (2021) [28]
	4.7 (0.97) Mean (SD)	2.9 (1.10)	<0.001		Novellis et al. (2018) [12]
Operative Time	3.5–4.5 h	2.86–4.23 h	<0.01	RATS results in longer operative time statistical significant in most trials	Oh et al. (2017) [22]
	215 min (median)	183 min (median)	0.0362		Augustin et al. (2013) [20]
	223 min (median)	202 min (median)	0.045		Deen et al. (2014) [21]
	241.7 min (median)	214.4 min (median)	0.06		BRAVO Trial (2022) [26]
Conversion to Thoracotomy	6.3%	13.1%	<0.01	Lower conversion rates were observed at RATS compared to VATS	Oh et al. (2017) [22]
	0% conversion rate	5.9% conversion rate	<0.05		Qiu et al. (2020) [25]
	19.2%	8.4%	0.4189		Augustin et al. (2013) [20]
	13.1%	20.5%	0.029		BRAVO Trial (2022) [26]
Postoperative Pain	0	1	1	No significant difference in postoperative pain perception (VAS score > 2) between groups at 3 or 30 days post-surgery	BRAVO Trial (2022) [26]
Hospital Stay	Median stay: 4–5 days	Median stay: 5–6 days	<0.006	Fewer hospital stay at patients underwent RATS compared to VATS (significant in most trials)	Oh et al. (2017) [22]
	Median stay: 5 days	Median stay: 6 days	<0.001		Novellis et al. (2018) [12]
	IQR = 2–5 days	IQR = 3–6 days	<0.001		Louie et al. (2016) [24]
	Median stay: 4 days	Median stay: 4 days	(0.6131)		Swanson et al. (2014) [19]
Postoperative Complications	34.1%	37.3%	<0.01	RATS shows fewer complications overall and in particular in bleeding and myocardial infarction events.	Oh et al. (2017) [22]
	18.9%	35.9%	0.12	RATS associated with fewer 90-day complications (not statistically significant)	BRAVO Trial (2022) [26]
Costs	USD 22,582 (median cost)	USD 17,874 (median cost)	<0.05	RATS as a technique costs higher amount compared to VATS at all trials measured	Paul et al. (2014) [34]
	USD 25,040.70 (average inpatient cost)	USD 20,476.60 (average inpatient cost)	<0.0001		Swanson et al. (2014) [19]
	USD 12,821 (hospitalization cost)	USD 8009 (hospitalization cost)	<0.001		RVlob Trial (2022) [31]
Readmission Rates	2.7%	20.5%	0.029	Significantly lower 90-day readmission rates compared to	BRAVO Trial (2022) [26]
	0%	16%	0.08	RATS associated with a trend toward reduced readmission rates, though not statistically significant.	ROMAN Trial (2021) [28]

RATS: Robotic-Assisted Thoracoscopic Surgery, VATS: Video-Assisted Thoracoscopic Surgery, IQR: Interquartile Range, SD: Standard Deviation.

## 4. Discussion

Robotic-assisted techniques show greater superiority over video-assisted techniques in many intraoperative parameters, especially those related to lymph node dissection [LND]. RATS does perform a greater extent of LND with statistical significance [12,28,31], and R0 resection is more achievable in lung cancer due to the completeness and accuracy of LND via the robotic-assisted method (30). This is crucial because any early-stage cancer surgery aims to achieve R0 resection as the definitive treatment. There is an ongoing debate on how the increased lymph node dissection, which is automatically linked to a more precise nodal upstaging, affects the overall survival and recurrent rates [32]. Although RATS results in a higher nodal yield, existing trials, such as BRAVO and ROMAN, have not identified any substantial survival benefits. Further prospective, long-term studies with standardized follow-up are required to confirm this hypothesis and ascertain whether the improved staging accuracy results in an improvement in disease-free and overall survival (DFS/OS).

Early-stage NSCLC includes stages IA, IB, IIA, and IIB (American Joint Committee on Cancer [AJCC] 7th edition), but the staging criteria were not consistently reported across the included studies, therefore possibly producing bias in outcomes such as lymph node dissection and survival rates. RATS may have more clear benefits in obtaining more comprehensive lymph node dissection and precise staging for stage II tumors, where nodal upstaging is more likely. On the other hand, VATS is a more affordable choice for stage IA and IB tumors without lymph node involvement. Most studies grouped early-stage NSCLC together without evaluating stage-specific outcomes, therefore making it challenging to determine the actual advantages of each method. Especially in terms of nodal upstaging and long-term survival, future studies should stratify patients by stage to adequately assess the efficacy of RATS and VATS.

Regarding postoperative complications and mortality, the safety profiles of RATS and VATS are comparable. Patients who underwent RATS had a shortened hospital duration of 2–5 days compared to 3–6 days for VATS [12,22,24], and the majority of studies have indicated that there is no significant difference between the two techniques in terms of postoperative mortality rates [22,27,31]. Faster recovery rates may result in increased patient satisfaction and reduced utilization of healthcare resources. RATS also shows lower conversion rates to thoracotomy, 6.3% versus 13.1% for VATS with statistical significance [22]. The procedure’s overall safety profile improves, and intraoperative morbidity decreases because of this reduced conversion rate [12,25]. Pain reduction and overall well-being data appear to favor RATS, as patients who undergo RATS procedures often require less postoperative opioid pain medication and return to their daily activities faster [36]. These advantages highlight the benefits that RATS has for minimizing surgical trauma. However, the findings regarding postoperative pain and quality of life are controversial due to the limited number of studies that address these outcomes. Most studies that are currently available do not include standardized pain assessments or long-term follow-up. Larger trials that employ standardized pain assessments are required to reach more definitive conclusions.

The absence of standardized criteria across studies complicates direct comparisons between RATS and VATS. There have been few RCTs so far; hence, there is a need to undertake more research to address this gap. These comparisons represent a challenge both in study design and in patient selection. In most cases, the choice between RATS and VATS is left to the operating surgeon. The choice of the best strategy must also be based on the unique characteristics of each patient, the surgeon’s expertise, and the capacity of the hospital. Yet, there is no established, standardized decision-making process to direct the selection of patients. To improve patient outcomes, future research should focus on developing a structured approach to selecting between RATS and VATS, considering factors like tumor complexity, stage distribution, patient risk profile, comorbidities, and institutional resources.

Furthermore, the differences in surgeon experience are another significant confounder in studies comparing RATS and VATS. High-volume surgeons tend to achieve better outcomes. The lack of adjustment for surgeon experience limits the validity of current findings. The comfort and familiarity of surgeons with robotic technology vary greatly and may influence recommendations for RATS. Surgeons who have more experience in open anatomic resection may find the VATS technique more conceptually intuitive. The data suggests that both VATS and RATS surgeons are likely to maintain a preference for one approach over another, as most RATS surgeons do not begin using a full robotic console until they believe that robotic surgery is superior to VATS in a specific case. Future studies should control for this variable to improve result reliability.

The widespread usage of RATS is mainly halted by its significantly higher costs [19,20,31,34,37,38]. The increase in cost comes from the substantial investment in capital, maintenance, and expendable parts associated with the robotic systems. The high cost combined with the limited availability of trained robotic specialists restricts RATS implementation. In areas that are limited in resources, this financial burden is quite substantial, which also requires a proper analysis of cost-effectiveness, especially with regard to the patient’s long-term benefits. Though RATS’s initial costs are significantly higher than VATS’s, the potential of lower readmission rates, shorter hospital stays, and reduced postoperative complications may offset these expenses. The accurate financial impact of RATS requires a full-cycle cost-effectiveness model including both direct (capital expenditure, disposable instruments, and maintenance) and indirect costs (postoperative care, complications management, rehabilitation cycle, and hospital readmission). This model would provide a more holistic assessment of the long-term cost-effectiveness of robotic-assisted surgery for early-stage NSCLC by comparing the initial investment to the prospective healthcare cost savings.

Another limitation of RATS is its longer operative time compared to VATS [20,21,23,24,26]. While the extended operative time reflects the meticulous dissection capabilities of robotic systems, it may also introduce logistical challenges in high-volume surgical centers, where operating room efficiency is paramount. Most trials comparing VATS and RATS for early-stage NSCLC are retrospective, using prospectively collected data, and a small number have focused solely on clinical outcomes and costs. The reliance on retrospective studies makes the findings prone to selection bias. Retrospective studies also limit the findings by lacking sensitivity analyses. Future research should include prospective RCTs with sensitivity and subgroup analyses to ensure more robust and reliable conclusions.

## 5. Conclusions

The present review represents the comprehensive study of the literature regarding the comparison of the perioperative outcomes between RATS and VATS for treating early-stage NSCLC. Evidence shows that minimally invasive techniques for early-stage NSCLC have optimal outcomes, with low perioperative mortality, minimum morbidity, and excellent long-term survival rates. While VATS and RATS are acknowledged as viable alternatives to open thoracotomy for the treatment of early-stage NSCLC, the optimal minimally invasive technique is still a topic of debate. Comparing RATS with VATS is essential in a time of increased early-stage lung cancer diagnosis due to stage migration [51]. Our results show that RATS achieves higher nodal upstaging. More research is required to find if this leads to better long-term outcomes. Oncological outcomes, including nodal upstaging, local recurrence, and survival, have yet to be properly assessed with sufficient long-term data. We need high-quality RCTs with longer follow-up periods and a larger patient population to compare the cost-effectiveness and oncologic outcomes of RATS and VATS. Prospective research on long-term financial results including surgical complications, hospital stay length, rehabilitation, and readmission rates is needed. Such studies would reveal the true cost of these different surgical approaches, allowing medical providers to choose the most cost-effective early-stage NSCLC treatment. At this point, it is unclear whether the RATS approach is a better option than VATS in the treatment of early-stage NSCLC, although it is certainly promising.

## Abbreviations

The following abbreviations are used in this manuscript:

RATS	Robotic assisted thoracoscopic surgery
VATS	Video assisted thoracoscopic surgery
NSCLC	Non-small cell lung cancer
SCLC	Small cell lung cancer
RCT	Randomized clinical trial
LND	Lymph node dissection
OR	Odds ratio
HR	Hazard ratio
SMD	Standardized mean difference
CI	Confidence interval
RFS	Relapse free survival
DFS	Disease free survival
OS	Overall survival
IQR	Interquartile range
AJCC	American Joint Committee on Cancer
